# Rethinking the eidos, genos, and diaphora of the health utility concept: a psychological perspective

**DOI:** 10.3389/fpsyg.2023.1139931

**Published:** 2023-06-19

**Authors:** Damien S. E. Broekharst, Sjaak Bloem, Edward A. G. Groenland, W. Fred van Raaij, Patrick P. T. Jeurissen, Michel van Agthoven

**Affiliations:** ^1^Center for Marketing and Supply Chain Management, Nyenrode Business University, Breukelen, UT, Netherlands; ^2^Department of Social Psychology, Tilburg University, Tilburg, NB, Netherlands; ^3^IQ Healthcare, Radboud University Medical Center, Nijmegen, GE, Netherlands; ^4^Janssen-Cilag B.V., Johnson & Johnson, Breda, NB, Netherlands

**Keywords:** health utility, health-state utility, utility, classic utility, expected utility, experienced utility, utilitarianism

## Abstract

The notion of utility gained a strong foothold in health economics over the last decades. However, the concept of health utility has not yet been decisively or irrefutably defined and the definitions that exist often do not take into account the current state of psychological literature. This perspective paper shows that the current definition of health utility emphasizes decision-making processes, deploys personal preferences, assumes psychological egoism, and attempts to objectively and cardinally measure utility. However, these foundational axioms that underly the current definition of health utility are not necessarily in concurrence with the current state of psychological literature. Due to these perceived shortcomings of the current health utility definition, it may be beneficial to redefine the concept of health utility in accordance with the current state of psychological literature. In order to develop such a revised definition of health utility the commonly deployed formula (Eidos = Genos + Diaphora) originating from Aristotle’s metaphysics is applied. The revised definition of health utility proposed in this perspective paper alludes to health utility as ‘the subjective value, expressed in terms of perceived pain or pleasure, that is attributed to the cognitive, affective and conative experience of one’s own physical, mental and social health state, which is determined through self-reflection and interaction with significant others’. Although this revised definition does neither replace nor supersede other conceptualizations of health utility, it may serve as a refreshing avenue for further discussion and could, eventually, support policymakers and health economists in operationalizing and measuring health utility in an even more accurate and veracious manner.

## 1. Introduction

Until the 1970s, the necessity of economic evaluation in healthcare was neither recognized nor desired as its insistence on attributing monetary value to human life was deemed unethical (Broome, 1991; Bentham, 1996; Berridge and O’Doherty, 2013; Jakovljevic and Ogura, 2016). However, by the 1980s, the support for economic evaluation in healthcare had gradually increased due to rising costs and lacking resources in healthcare generated by economic crisis, the advent of new and expensive treatments, and the general increase in lifespan (Broome, 1991; Bentham, 1996; Berridge and O’Doherty, 2013; Jakovljevic and Ogura, 2016). As health economics was still in its infancy by then, there were no specific methods for economic evaluation in healthcare available yet, prompting the adoption and adjustment of existing methods (e.g., standard gamble approach, time trade-off method) from other economic fields in which economic evaluation was already well established (e.g., welfare economics, labor economics) (Broome, 1991; Bentham, 1996; Berridge and O’Doherty, 2013; Jakovljevic and Ogura, 2016). With the introduction of these methods, the notion of utility gained a strong foothold in the field of health economics as it serves as a central concept in health economic evaluation (Broome, 1991; Bentham, 1996; Berridge and O’Doherty, 2013; Jakovljevic and Ogura, 2016). However, as the concept of utility originated in other fields of economics and was introduced for mostly pragmatic reasons, the concept of utility in the context of healthcare, also known as health utility, has not yet been decisively or irrefutably defined and the provisory definitions that do exist are often without consideration of the current state of psychological literature (Broome, 1991; Bentham, 1996; Berridge and O’Doherty, 2013; Jakovljevic and Ogura, 2016). Therefore, this perspective paper attempts to (1) clarify the historical evolution regarding the definition of health utility, (2) explicate the theoretical criticism concerning the current definition of health utility, and (3) redefine the definition of health utility in accordance with the current state of psychological literature. Although this revised definition does neither replace nor supersede other conceptualizations of health utility, it may serve as a refreshing avenue for further discussion and could, eventually, support policymakers and health economists in operationalizing and measuring health utility in an even more accurate and veracious manner.

## 2. Historical evolution

Over the past centuries, the definition of utility has been subject to persistent debate and continual reconfiguration among a variety of different scientific fields (Broome, 1991; Bentham, 1996; Berridge and O’Doherty, 2013). This discussion concerning the definition of utility was especially vibrant in and between the scientific fields of philosophy, economics, and psychology (Broome, 1991; Bentham, 1996; Berridge and O’Doherty, 2013).

### 2.1. The definition of classic utility

The first rudimentary proto-definitions of utility were conceived within the British Moralist tradition by the likes of Richard Cumberland, Francis Hutcheson, John Gay, and David Hume (Broome, 1991; Bentham, 1996). However, the first formal and detailed definition of utility as a concept emerged from the utilitarian tradition and was established by utilitarian thinkers such as Jeremy Bentham, John Stuart Mill, and their contemporaries (Broome, 1991; Bentham, 1996). The definition established by Bentham, Mill, and their fellow utilitarian thinkers indicates that utility constitutes the property in any action or object, whereby it tends to produce benefit, advantage, pleasure, good, or happiness or to prevent the happening of mischief, pain, evil, or unhappiness to the party whose interest is considered (Broome, 1991). This definition seems to focus on the concept of utility as the outcome of an action, event or decision without clarifying the mechanism through which that particular outcome is established revealing the consequentialist fundamentals underlying this definition (Broome, 1991; Bentham, 1996; Berridge and O’Doherty, 2013). This definition also seems to specifically explicate the utility of an outcome in terms of the promulgation of pleasure and the preclusion of pain indicating an inherently hedonist perspective underlying this definition (Broome, 1991; Bentham, 1996; Berridge and O’Doherty, 2013). This definition further seems to imply that the promulgation of pleasure and the preclusion of pain only concerns the party whose interest is considered emphasizing the Hobbesian notion of psychological egoism underlying this definition (Broome, 1991; Bentham, 1996; Berridge and O’Doherty, 2013). This definition, moreover, only seems to consider the quantity of pleasure and pain without taking into account the existence of higher and lower pleasures exposing the egalitarian principles and cardinalist tendencies underlying this definition (Broome, 1991; Bentham, 1996; Berridge and O’Doherty, 2013). Although the definition established by Bentham, Mill, and their fellow utilitarian thinkers came under increasing scrutiny in later years, it remained relatively untarnished and unaltered until the conception and divergence of the modern scientific field of economics (Berridge and O’Doherty, 2013).

### 2.2. The definition of economic utility

The notion of utility was introduced in the field of economics by the likes of William Stanley Jevons, Carl Menger, and Léon Walras during the 1870s, which resulted in a fierce debate between many factions within economic discourse (e.g., marginalists, ordinalists, and cardinalists) about its measurability and its relation to risk and uncertainty (von Neumann and Morgenstern, 1944; Caplin and Leahy, 2001). These apparent theoretical conflicts prevented the development of a widely accepted definition of utility in the field of economics. It was only during the 1940s that a somewhat acceptable theory was put forward by John von Neumann and Oskar Morgenstern, which was, although hesitantly, tolerated in certain fields of economics and allowed for the embedding of a more excogitated definition (von Neumann and Morgenstern, 1944; Caplin and Leahy, 2001). The definition of utility put forward by Neumann and Morgenstern indicates that utility is a weighted average of the utilities of each of its possible outcomes, where the utility of an outcome measures the extent to which that outcome is preferred, or preferable, to the alternatives (von Neumann and Morgenstern, 1944; Caplin and Leahy, 2001). The utility of each outcome is weighted according to the probability that the act will lead to that outcome (Schoemaker, 1982; Kahneman and Thaler, 1991). This definition seems to focus on the concept of utility as the decision-making process that generates a certain outcome without explicating the nature of that outcome revealing the deontological fundamentals underlying this definition, which contrasts the definition of classic utility that prioritizes the explanation of actual outcomes (Schoemaker, 1982; Kahneman and Thaler, 1991). This definition subsequently seems to explicate the decision-making process in terms of establishing, weighing, and pursuing personal preferences indicating the preference utilitarianist principles underlying this definition, which is also unlike the definition of classic utility that emphasizes hedonic experience (Schoemaker, 1982; Kahneman and Thaler, 1991). This definition further seems to imply that establishing, weighing, and pursuing personal preferences in order to optimize the utility of a certain outcome is a highly individualized and ultimately self-interested process emphasizing the Hobbesian notion of psychological egoism underlying this definition, which is similar to the definition of classic utility (Briggs, 2014). This definition, moreover, only seems concerned with quantifying the utility of an outcome based on predicted probability levels showing the cardinalist nature of this definition, which is also present in the definition of classic utility albeit less pronounced (von Neumann and Morgenstern, 1944; Schoemaker, 1982; Kahneman and Thaler, 1991; Caplin and Leahy, 2001; Briggs, 2014). Although the aforementioned definition of economic utility is the product of an uneasy compromise, it still permeated throughout the field of health economics (von Neumann and Morgenstern, 1944; Schoemaker, 1982; Kahneman and Thaler, 1991; Caplin and Leahy, 2001; Briggs, 2014).

### 2.3. The definition of health utility

In the early 1970s, health economists by the likes of George Torrance and his contemporaries introduced the concepts of health utility in the field of health economics (Torrance et al., 1982; Tolley, 2009). Health utility is obtained through a process of health state measurement followed by subsequent health state valuation and is deployed in order to value the health outcomes attained after performing certain health interventions (Tolley, 2009; Wolowacz et al., 2016). The definition of health utility proposed by George Torrance and his contemporaries refers to a representation of weighed preference for a given health-related outcome on a cardinal numerical scale, where a value of 1.0 represents full health, 0.0 represents dead, and negative values represent states worse than dead (Torrance et al., 1982; Wolowacz et al., 2016). This definition seems to describe the concept of health utility as the decision-making process that produces a certain health outcome without clarifying the nature of that health outcome displaying the deontological fundamentals underlying this definition, which is akin to the definition of economic utility (Torrance et al., 1982; Wolowacz et al., 2016). This definition also seems to describe the decision-making process in terms of establishing, weighing, and pursuing personal health preferences indicating the preference utilitarianist principles underlying this definition, which is also similar to the definition of economic utility (Torrance et al., 1982; Wolowacz et al., 2016). This definition further seems to imply that establishing, weighing, and pursuing personal health preferences in order to optimize health utility is a highly individualized and ultimately self-interested process emphasizing the Hobbesian notion of psychological egoism underlying this definition, which is also present in the definition of economic utility (Torrance et al., 1972). This definition, moreover, seems mostly concerned with quantifying health utility based on a numerical scale showing the cardinalist nature of this definition, which is also put forward in the definition of economic utility albeit differently and less explicitly operationalized (Torrance et al., 1972, 1982; Kahneman et al., 1997; Tolley, 2009; Wolowacz et al., 2016). This definition of health utility and its particular characteristics shows strong resemblance with the definition of the economic utility concept described earlier as is displayed in Table 1 (Torrance et al., 1972, 1982; Kahneman et al., 1997; Tolley, 2009; Wolowacz et al., 2016). Although the aforementioned definition of utility is still most prominent throughout the field of health economics, its foundational axioms are not necessarily in concurrence with the current state of psychological literature (Torrance et al., 1972, 1982; Kahneman et al., 1997; Tolley, 2009; Wolowacz et al., 2016).

**Table 1:** Comparison of utility definitions.

	Classic utility	Economic utility	Health utility
*Descriptive focus*	Decision-making outcome	Decision-making process	Decision-making process
*Appraisal approach*	Hedonic experience	General preference	Health preference
*Human motives*	Psychological egoism	Psychological egoism	Psychological egoism
*Measurement method*	Cardinal	Cardinal using probability	Cardinal using numeric scale

## 3. Critical perspectives

An ever increasing contingent of psychologists and behavioral economists, led by the likes of Daniel Kahneman, Amos Tversky, and Richard Thaler, criticized the aforementioned definition of health utility as they find fault with, most especially, its emphasis on decision-making processes, its deployment of personal preferences, its assumption of psychological egoism, and its attempt to objectively and cardinally measure utility (Kahneman and Thaler, 1991; Kahneman et al., 1997). Many authors argued that the current definition of health utility fails to fulfill its basic function as this definition puts emphasis on the decision-making process that generates the health utility without providing a clear and concise description of its particular essence, intrinsic nature, and indispensable qualities (Kahneman and Tversky, 1979; Kahneman and Thaler, 1991; Kahneman et al., 1997; Kahneman and Sugden, 2005). Several authors also stated that the current definition of health utility makes gratuitous use of personal preferences in order to approximate actual experienced utility as research shows that personal preferences are unstable over time and are based on incomplete information as well as bounded rationality making them inappropriate for projective purposes (Kahneman and Tversky, 1979; Simon, 1990; Kahneman and Sugden, 2005; Schilirò, 2018). Most authors further argued that the current definition of health utility unjustifiably adopts the premise of psychological egoism as research suggests that certain human behavior does not seem to be explained by self-regarding desires and posits that one must desire things beyond one’s own self-regarding desires in order to actualize them (Kahneman and Tversky, 1979; Kahneman and Thaler, 1991; Kahneman et al., 1997; Kahneman and Sugden, 2005). Many authors, moreover, argued that the current definition of health utility unrealistically attempts to objectively and cardinally measure utility as research shows that the utility ascribed to a health intervention is inherently a product of subjective experiences making it difficult to cardinally compare, add, or subtract utility (Kahneman and Tversky, 1979; Kahneman and Thaler, 1991; Kahneman et al., 1997; Kahneman and Sugden, 2005). Due to the perceived shortcomings of the current health utility definition, these psychologists and behavioral economists posit that it may be beneficial to redefine the concept of utility in health economics in accordance with the current state of psychological literature (Kahneman and Tversky, 1979; Kahneman and Thaler, 1991; Kahneman et al., 1997; Kahneman and Sugden, 2005).

## 4. New definition

In the remainder of this perspective paper, a rudimentary attempt is levied at revising the definition of health utility in accordance with the current state of psychological literature. The revision method, revision process and revised definition will be sequentially presented in order to provide the necessary procedural transparency and substantiate the final result.

### 4.1. The revision method

In order to develop such a revised definition of health utility a commonly deployed formula originating from Aristotle’s metaphysics is applied as it is one of the few methods that allows for the logical, structured and modular development of complex definitions composed of nested sub-definitions (Smith, 2020; Cohen and Reeve, 2021). This conceptual formula conceives of a definition by determining and integrating three core elements, namely eidos (εἶδος), genos (γένος) and diaphora (διαφορά) (Smith, 2020; Cohen and Reeve, 2021). Eidos refers to the particular essence, intrinsic nature, and indispensable quality of a certain concept (Smith, 2020; Cohen and Reeve, 2021). Genos refers to a class of concepts that share common characteristics to which a certain concept belongs (Smith, 2020; Cohen and Reeve, 2021). Diaphora refers to the particular dimensions, elements, or attributes that distinguish a particular concept from other concepts belonging to the same genos (Smith, 2020; Cohen and Reeve, 2021). This formula posits that eidos can be captured by stating its genos and diaphora as is depicted below (Smith, 2020; Cohen and Reeve, 2021).

Eidos=Genos+Diaphora

Although this formula provides definitions with sufficient perspicuity, it should be mentioned that the definition of more complex concepts may contain certain subordinate concepts that need some additional explication in their own right (Smith, 2020; Cohen and Reeve, 2021).

### 4.2. The revision process

In this perspective paper, health utility can be considered the concept of which the essence, intrinsic nature, and indispensable qualities needs to be captured in a comprehensive definition (eidos) (Smith, 2020; Cohen and Reeve, 2021). This concept of health utility is commonly classified as a manner to express subjective value alongside other expressions of value belonging to the same class, such as acquisition utility, transaction utility, and procedural utility (genos; Smith, 2020; Cohen and Reeve, 2021). This expression of subjective value in the case of health utility is distinguished from other expressions of subjective value as it pertains especially to the experience of one’s own health state (diaphora; Smith, 2020; Cohen and Reeve, 2021). The aforementioned classification and specific attributes of health utility introduce three subordinate concepts, namely value, experience and health state, that may need some additional explication in order to clarify the eventual definition. In contemporaneous scientific literature two types of subjective value are distinguished, *videlicet*, utilitarian and hedonic value (Khare, 2011; Camp et al., 2017). Utilitarian value refers to the personal and rational comparison of sacrifices and benefits based on which a preference for a certain outcome is established, while hedonic value refers to the pain and pleasure individuals derive from experiencing a particular outcome (Khare, 2011; Camp et al., 2017). As this perspective paper already discussed that utilitarian value and its emphasis on preferences has been confronted with considerable theoretical criticism, the deployment of hedonic value and its emphasis on experienced pain and pleasure seems more appropriate (Khare, 2011; Camp et al., 2017). Therefore, in the revised definition of health utility the concept of value could be explicated in terms of the pleasure and pain derived from a particular outcome. Furthermore, in current scientific literature human experience is often explicated using a psychological triad consisting of cognitive, affective and conative perceptions established through self-reflection and interaction with significant others (Dennis et al., 2013). Cognition refers to the process of acquiring and comprehending knowledge (Dennis et al., 2013). Affection refers to process of feeling and processing emotions (Dennis et al., 2013). Conation refers to process of performing and accomplishing actions (Dennis et al., 2013). As this longstanding psychological triad constitutes a consistently valid and reliable operationalization of human experience, its deployment seems suitable (Dennis et al., 2013). Therefore, in the revised definition of health utility the concept of human experience could be explicated in terms of cognitive, affective and conative perceptions established through self-reflection and interaction with significant others. Moreover, in contemporary scientific literature countless attempts have been levied at identifying the core elements of general or disease-specific health states resulting in a myriad of health state taxonomies and typologies (Callahan, 1973; Bloem and Stalpers, 2012; Bloem et al., 2020). Although the content of these health state taxonomies and typologies may differ research shows that three basic elements underly the health state concept, namely a physical, mental and social element (Callahan, 1973; Bloem and Stalpers, 2012; Bloem et al., 2020). This tripartite model is a widely acknowledged and commonly deployed operationalization that shows validity and reliability across populations and countries (Callahan, 1973; Bloem and Stalpers, 2012; Bloem et al., 2020). Therefore, in the revised definition of health utility the concept of health state could be explicated in terms of a physical, mental and social element.

### 4.3. The revised definition

**Figure 1:**
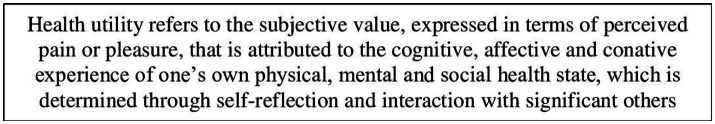
Revised definition of health utility.

Given the aforementioned consideration and clarifications, a revised definition of health utility has been established, which is displayed in Figure 1.

Although this revised definition does neither replace nor supersede other conceptualizations of health utility, it may serve as a refreshing avenue for further discussion and could, eventually, support policymakers and health economists in operationalizing and measuring health utility in an even more accurate and veracious manner.

## 5. Discussion

This perspective paper levied a rudimentary attempt at redefining the concept of health utility in accordance with the current state of psychological literature, eventually, suggesting a revised definition of health utility. This revised definition provides a detailed description of health utility as well as a further explication of its constituent elements (i.e., value, experience, and health state) avoiding excessive focus on decision-making processes or methodological procedures (Kahneman and Thaler, 1991; Kahneman et al., 1997). This revised definition also emphasizes the individual health experiences that are central to the determination of health utility avoiding the deployment of health preferences as unnecessary proxies (Kahneman and Thaler, 1991; Kahneman et al., 1997). This revised definition further states that these individual health experiences are established through self-reflection and interaction with significant others rejecting the Hobbesian notion of psychological egoism and recognizing the altruistic tendencies present in human psychology (Kahneman and Thaler, 1991; Kahneman et al., 1997). This revised definition, moreover, presents health utility as the subjective value attributed to individual health experiences rejecting the notion of objective and cardinal measurement and endorsing a more ordinalist perspective on the determination and expression of health utility (Kahneman and Thaler, 1991; Kahneman et al., 1997). By integrating these four foundational axioms in the revised definition of health utility, the most important criticisms levied against the current definition of health utility have been accommodated. Although the revised definition of health utility proposed in this perspective paper has been thoroughly contemplated, it should be mentioned that alternative explications of this revised definition and its constituent elements might also be considered. For instance, the concept of value is explicated in terms of perceived pain and pleasure, while one might suggest that a more economic or monetary explication of value (e.g., quality-adjusted life years, willingness to pay) could be more prudent as the concept of health utility is central to health economic evaluation (Whitehead and Shehzad, 2010). Similarly, the concept of human experience is explicated in terms of cognitive, affective, and conative perceptions, while one might argue that other typologies of human experience (e.g., sensory, intellectual, and behavioral) could also be appropriate (Buccini and Padovani, n.d.). Likewise, the concept of health state is explicated in terms of a physical, mental, and social element, while one might suggest that this explication lacks comprehensibility and needs to be expanded with other important elements (e.g., spirituality) or that a more detailed disquisition of these elements is necessary (e.g., ambulation, self-care, discomfort, depression, fatigue, hearing, and vision; Geraerds et al., 2021). Regardless of these considerations, it seems apparent that this revised definition may have considerable practical implications for health economic evaluation and subsequent health policy as one might argue that the operationalization and measurement of a health utility concept that is more in accordance with the current state of psychological literature could eventually generate health economic evaluation and subsequent health policy that is more in concordance with the actual health experiences of patients and the public. However, in order to accomplish this challenging endeavor, at least, two avenues for future research should be pursued. The first avenue is concerned with developing an appropriate operationalization of the revised definition in collaboration with experts, healthcare professionals, patients and the public as further explication of its core components might be appropriate. The second avenue is concerned with developing an ordinal measurement method (e.g., visual analog scales, self-anchored ladder scales) for examining the revised definition as it rejects objective and cardinal measurement. This perspective paper appeals to the scientific community to resolve these difficult issues in order to support policymakers and health economists in operationalizing and measuring health utility in an even more accurate and veracious manner.

## Data availability statement

The original contributions presented in the study are included in the article/supplementary material; further inquiries can be directed to the corresponding author.

## Author contributions

DB wrote the manuscript. SB and EG supervised the research project. SB, EG, WR, PJ, and MA reviewed and edited the manuscript. All authors contributed to the article and approved the submitted version.

## Funding

This work was supported by Janssen-Cilag B.V. The funder was not involved in the study design, collection, analysis, interpretation of data, the writing of this article or the decision to submit it for publication.

## Conflict of interest

MA was employed by Janssen-Cilag B.V., Johnson & Johnson.

The remaining authors declare that the research was conducted in the absence of any commercial or financial relationships that could be construed as a potential conflict of interest.

## Publisher’s note

All claims expressed in this article are solely those of the authors and do not necessarily represent those of their affiliated organizations, or those of the publisher, the editors and the reviewers. Any product that may be evaluated in this article, or claim that may be made by its manufacturer, is not guaranteed or endorsed by the publisher.
